# The efficacy of medical masks and respirators against respiratory infection in healthcare workers

**DOI:** 10.1111/irv.12474

**Published:** 2017-08-30

**Authors:** Chandini Raina MacIntyre, Abrar Ahmad Chughtai, Bayzidur Rahman, Yang Peng, Yi Zhang, Holly Seale, Xiaoli Wang, Quanyi Wang

**Affiliations:** ^1^ School of Public Health and Community Medicine University of New South Wales Sydney NSW Australia; ^2^ College of Public Service & Community Solutions and College of Health Solutions Arizona State University Phoenix AZ USA; ^3^ The Beijing Centre for Disease Prevention and Control Beijing China

**Keywords:** droplet infections, healthcare workers, influenza, masks, medical masks, respirators

## Abstract

**Objective:**

We aimed to examine the efficacy of medical masks and respirators in protecting against respiratory infections using pooled data from two homogenous randomised control clinical trials (RCTs).

**Methods:**

The data collected on 3591 subjects in two similar RCTs conducted in Beijing, China, which examined the same infection outcomes, were pooled. Four interventions were compared: (i) continuous N95 respirator use, (ii) targeted N95 respirator use, (iii) medical mask use and (iv) control arm. The outcomes were laboratory‐confirmed viral respiratory infection, influenza A or B, laboratory‐confirmed bacterial colonisation and pathogens grouped by mode of transmission.

**Results:**

Rates of all outcomes were consistently lower in the continuous N95 and/or targeted N95 arms. In adjusted analysis, rates of laboratory‐confirmed bacterial colonisation (RR 0.33, 95% CI 0.21‐0.51), laboratory‐confirmed viral infections (RR 0.46, 95% CI 0.23‐0.91) and droplet‐transmitted infections (RR 0.26, 95% CI 0.16‐0.42) were significantly lower in the continuous N95 arm. Laboratory‐confirmed influenza was also lowest in the continuous N95 arm (RR 0.34, 95% CI 0.10‐1.11), but the difference was not statistically significant. Rates of laboratory‐confirmed bacterial colonisation (RR 0.54, 95% CI 0.33‐0.87) and droplet‐transmitted infections (RR 0.43, 95% CI 0.25‐0.72) were also lower in the targeted N95 arm, but not in medical mask arm.

**Conclusion:**

The results suggest that the classification of infections into droplet versus airborne transmission is an oversimplification. Most guidelines recommend masks for infections spread by droplets. N95 respirators, as “airborne precautions,” provide superior protection for droplet‐transmitted infections. To ensure the occupational health and safety of healthcare worker, the superiority of respirators in preventing respiratory infections should be reflected in infection control guidelines.

## 1 BACKGROUND

There is currently a lack of consensus around the efficacy of medical masks and respirators for healthcare workers (HCWs) against influenza, with only five published randomised control trials (RCTs) in HCWs conducted to date.^1^, ^2^, ^3^, ^4^, ^5^ While N95 respirators have been shown to be superior to medical masks in preventing clinical respiratory infection (CRI), influenza illness (ILI) and other outcomes, none of the studies were adequately powered to examine laboratory‐confirmed influenza.

In the smallest of the trials, involving only 32 HCWs, there was no difference in the rates of respiratory illnesses between HCWs who used medical masks and the control group.^1^ A Canadian study of 422 hospital nurses compared targeted use of N95 respirators and medical masks and found that the rate of serologically defined influenza was 25% in both arms.^2^ However, in the absence of a control arm for comparison, the finding of no difference in outcomes between the intervention arms could represent either equal efficacy or equal inefficacy of the two interventions. The other two published HCW RCTs used a more specific and less sensitive definition of influenza based on nucleic acid testing (NAT) of respiratory specimens in symptomatic subjects. As such, even these substantially larger RCTs were unable to demonstrate any significant difference in influenza infection between N95 respirators and medical masks.^3^, ^4^ Finally, a recent study examined the efficacy of cloth masks compared to medical mask and control groups, and found that cloth masks may increase the risk of infection in HCWs.^5^

Guidelines for respiratory protection have been driven by presumed transmission mode alone, and under an assumption that influenza and other pathogens are spread by one mode alone.^6^ However, the paradigm of unimodal droplet or airborne spread is based on outmoded experiments from the 1940s, which concluded that only large droplets are found at close proximity to the patient, while small droplet nuclei and airborne particles are found at a longer distance.^7^, ^8^, ^9^ It has since been shown that both small and large particles can exist at short distances from the patient, and that aerosolised transmission can occur at close proximity.^9^

In our two published RCTs conducted in China,^3^, ^4^ we used the same outcomes, case definitions and measurement tools, and used the same testing methods for a range of different pathogens transmitted by different routes. This afforded an opportunity to pool the data from both trials for improved statistical power to examine the outcomes by pathogens and mode of transmission. The aim of this pooled analysis was to examine the efficacy of medical masks and respirators in HCWs against respiratory infection.

## 2 METHODS

We pooled the results of our two RCTs on mask and respirator use in hospital HCWs in Beijing, China. The first RCT (Trial 1) was conducted from December 2008 to January 2009,^3^ and included 1441 HCWs randomised to: medical mask arm (n = 492), N95 fit‐tested arm (n = 461) and N95 non‐fit‐tested arm (n = 488). The rate of fit‐test failure was very low (5/461) in this trial, so data from both N95 arms were combined for analysis.

An additional 481 healthcare workers from nine hospitals were recruited to a control arm. These hospitals were purposefully selected as they indicated low levels of routine mask/respirator use during a pre‐trial assessment. Participants in the control arms continued their usual mask wearing practices and were followed using the same protocol as applied to the other arms.^3^

The second trial (Trial 2) was conducted from 28 December 2009 to 7 February 2010, using the same design.^4^ In Trial 2, participants were randomised to three arms: medical masks at all times on shift (n = 572), continuous N95 respirators at all times on shift (n = 516) and targeted/intermittent use of N95 respirators only while doing high‐risk procedures or barrier nursing of a patient with known respiratory illness (n = 521). Fit testing was not performed in the second RCT. In both trials, participants were followed for 4 weeks of wearing the medical masks or respirators, and an extra week of non‐wearing of masks for the development of symptoms. Demographic and clinical data were collected, including gender, age, smoking, vaccination status, pre‐existing medical illnesses, hand hygiene and high‐risk procedures. Pharyngeal swabs were collected from symptomatic participants, and samples were tested at the laboratories of the Beijing Centers for Disease Control and Prevention. There was no major difference in the products used in both clinical trials. In the first trial, we used medical masks (3M, catalogue number 1820) and N95 fit/non‐fit‐tested respirator (3M, catalogue number 9132). The following products were used in the second trial: medical masks (3M, catalogue number 1817) and respirator (3M, catalogue number 1860).

The interventions compared in the pooled analysis were as follows: (i) continuous use of N95 respirators (pooled data from both trials ‐ 1530 subjects); (ii) targeted N95 respirator use (data from trial 2‐516 subjects); (iii) continuous use of medical masks (pooled data from both trials ‐ 1064 subjects) and (iv) and a control group (data from trial 1‐481 subjects).

Only laboratory‐confirmed outcomes were included in the analysis, which were defined and measured identically in both trials, and comprised: (i) laboratory‐confirmed viral respiratory infection (detection of adenoviruses; human metapneumovirus; coronavirus 229E ⁄ NL63; parainfluenza viruses 1, 2 and 3; influenza viruses A and B; respiratory syncytial virus A and B; rhinovirus A⁄B and coronavirus OC43 ⁄HKU1 by multiplex PCR); (ii) laboratory‐confirmed (multiplex PCR) influenza A or B and (iii) laboratory‐confirmed bacterial colonisation (Streptococcus pneumonia, Haemophilus influenza, Bordetella pertussis, Chlamydophila pneumoniae and Mycoplasma pneumonia).^3^, ^4^ The laboratory testing has previously been described.^3^, ^4^

Laboratory‐confirmed bacteria and viruses identified in participants were categorised according to droplet (n = 285), contact (n = 6) and airborne (n = 3) transmission modes (Table S1A). Sixty‐one co‐infection cases with multitransmission were categorised separately. Among the viruses isolated, coronavirus and influenza A/B were included in the droplet category (and thus included in the additional analysis); rhinovirus A/B was included in the airborne category and adenovirus; parainfluenza virus and respiratory syncytial virus (RSV) were included in contact category in the base case analysis. All bacteria were categorised into the droplet transmission category. For consistency, data on the transmission modes were taken from the Pathogen Safety Data Sheets (PSDSs) of the Public Health Agency of Canada^10^ (Table S1A). As the largest number of confirmed infections was in the droplet category, we conducted a subgroup analysis of droplet‐transmitted infections. Given there were a large number of RSV cases (n = 33) in our data set and RSV is variously categorised as either “droplet”^11^ or “contact” spread^12^ in different guidelines, we performed a sensitivity analysis by including RSV into the droplet transmission category instead of contact.

### 2.1 Ethics

Ethics approvals of two clinical trials were obtained from the Institutional Review Board and Human Research Ethics Committee of the Beijing Center for Disease Prevention and Control.

### 2.2 Patient involvement

We did not involve patients and their families in the design and conduct of the study. We have acknowledged the support of participants, and the results will be published in open access journal.

### 2.3 Statistical analysis

The data sets from the two trials were pooled incorporating the common variables. We calculated the attack rate (proportion of outcome) of each of the four outcomes by the study arms.

We conducted a fixed effect individual patient data (IPD) meta‐analysis by fitting a multivariable log binomial model, using generalised estimating equation (GEE) to account for clustering by hospital/ward. We used a fitted fixed effect model because there are only two trials. Two studies were conducted in the same setting with similar participant characteristics, and they examined the same underlying effect. In the analysis, relative risk (RR) was estimated using the control arm as the referent category after adjusting for potential confounders and their interaction terms with a trial ID number. The overall rates of seasonal infection were higher in the second trial than the first. The consistency assumption (ie between study homogeneity) for the IPD meta‐analysis was tested by fitting an interaction term between trial ID and trial arms where a significant interaction is indicative of inconsistency.^13^ Any interaction term (between trial ID and covariates other than trial arm) that was not a confounder was subsequently excluded from the model using backward elimination approach. This approach is described in detailed elsewhere.^4^ We repeated the above‐described methods for each of the outcomes.

## 3 RESULTS

**Figure 1:**
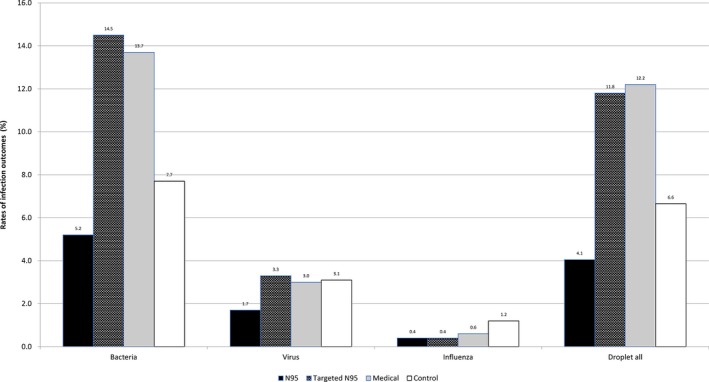
Rate of infections reported in HCWs in the different arms from Trials 1 and 2

After combining the data sets from the two trials, 3591 cases were entered into the pooled analysis (1064 cases in the medical mask arm, 516 cases in the targeted N95 arm, 1530 cases in the continuous N95 arm and 481 cases in the control arm). The infection outcomes are presented in Figure 1. The rates of laboratory‐confirmed viral respiratory infection (26/1530, 1.7%), laboratory‐confirmed bacterial colonisation (79/1530, 5.2%) and droplet‐transmitted infections (62/1530, 4.1%) were lowest among the continuous N95 arm. Laboratory‐confirmed influenza A and B was lowest in continuous N95 (6/1530, 0.4%) and targeted N95 arms (2/516, 0.4%).

In the IPD meta‐analysis, none of the interaction terms between trial arm and trial ID was significant for any of the outcome variables. Thus, the consistency assumption for the IPD meta‐analysis was satisfied. However, a significant interaction was observed between trial ID and hand washing for laboratory‐confirmed bacterial colonisation only; therefore, we estimated the RR for trial ID stratified by hand washing.

**Figure 2:**
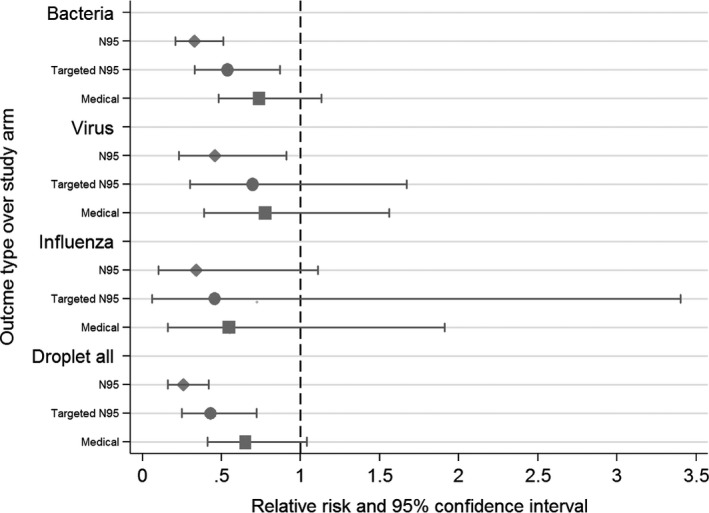
Forest plot of outcomes according to various interventions

Figure 2 shows the forest plot of outcomes according to various interventions. All outcomes were consistently lower in the continuous N95 and targeted N95 arms. The IPD meta‐analysis shows that the risk of laboratory‐confirmed bacterial colonisation was lower in the continuous N95 arm (RR 0.33, 95% CI 0.21‐0.51 or 67% efficacy) and targeted N95 arm (RR 0.54, 95% CI 0.33‐0.87 or 46% efficacy) (Table 1).

**Table 1:** Multivariable cluster adjusted log binomial model of laboratory‐confirmed bacterial colonisation

Variables in the model	Relative risk (95% CI)	*P*‐value
Continuous N95 arm	**0.33 (0.21‐0.51)**	**<.001**
Targeted N95 arm	**0.54 (0.33‐0.87)**	**.001**
Medical mask arm	0.74 (0.48**‐**1.13)	.161
Control arm	Ref	Ref
Sex (Male)	0.60 (0.42**‐**0.85)	.005
Trial	2.53 (1.65**‐**3.87)	<.001
Influenza vaccine	1.13 (0.89**‐**1.43)	.308
Trial * Hand wash	4.49 (3.12**‐**6.48)	<.001

Bold value indicates statistically significant results.

Laboratory‐confirmed viral respiratory infections were significantly lower in the continuous N95 arm (RR 0.46, 95% CI 0.23‐0.91, or 54% efficacy). The rates of laboratory‐confirmed virus were also lower in the targeted N95 arm (RR 0.70, 95% CI 0.30‐1.67) and medical masks arm (RR 0.78, 95% CI 0.39‐1.56); however, the difference was not statistically significant (Table 2).

**Table 2:** Multivariable cluster adjusted log binomial model of laboratory‐confirmed viral respiratory infection

Variables in the model	Relative risk (95% CI)	*P*‐value
Continuous N95 arm	**0.46 (0.23‐0.91)**	**.026**
Targeted N95 arm	0.70 (0.30‐1.67)	.424
Medical mask arm	0.78 (0.39‐1.56)	.484
Control arm	Ref	Ref
Sex (Male)	0.69 (0.36‐1.33)	.272
Hand washing	0.78 (0.51‐1.20)	.264
Influenza vaccine	0.94 (0.57‐1.55)	.808
Trial	1.50 (0.89‐2.54)	.131

Bold value indicates statistically significant results.

Laboratory‐confirmed influenza was also lowest in continuous N95 arm (RR 0.34, 95% CI 0.10‐1.11) but not significant (Table 3). In the subgroup analysis of droplet‐transmitted infections, compared to the control arm, the efficacy of continuous N95 respirators against droplet‐transmitted infections (bacterial and viral) was 74% (RR 0.26, 95% CI 0.16‐0.42) and 57% in the targeted N95 arm (RR 0.43, 95% CI 0.25‐0.72) (Table 4).

**Table 3:** Multivariable cluster adjusted log binomial model of laboratory‐confirmed influenza A or B

Variables in the model	Relative risk (95% CI)	*P*‐value
Continuous N95 arm	0.34 (0.10‐1.11)	.074
Targeted N95 arm	0.46 (0.06‐3.40)	.445
Medical mask arm	0.55 (0.16‐1.91)	.350
Control arm	Ref	Ref
Sex (Male)	0.27 (0.03‐2.01)	.220
Hand washing	0.70 (0.29‐1.73)	.446
Influenza vaccine	0.78 (0.26‐2.34)	.660
Trial	0.64 (0.19‐2.18)	.477

**Table 4:** Multivariable cluster adjusted log binomial model of droplet‐transmitted infections

Variables in the model	Relative risk (95% CI)	*P*‐value
Continuous N95 arm	**0.26 (0.16‐0.42)**	**<.001**
Targeted N95 arm	**0.43 (0.25‐0.72)**	**.001**
Medical mask arm	0.65 (0.41‐1.04)	.074
Control arm	Ref	Ref
Sex (Male)	0.63 (0.43‐0.92)	.016
Hand washing	1.27 (0.99‐1.62)	.068
Influenza vaccine	1.16 (0.90‐1.50)	.257
Trial	3.97 (2.83‐5.59)	<.001

Bold value indicates statistically significant results.

Inclusion of RSV cases in the droplet‐transmitted pathogen category did not change the risk ratio to a large extent. If RSV cases were also included in the droplet‐transmitted pathogen category, the efficacy was 70% in the continuous N95 (RR 0.30, 95% CI 0.19‐0.46) and 51% in the targeted N95 arms (RR 0.49, 95% CI 0.30‐0.80). The rate of droplet only transmitting viral infections was also lower in the continuous N95 and targeted N95 arms. HCWs who used a continuous N95 and targeted respirator were 85% (RR 0.15, 95% CI 0.04‐0. 59) and 84% (RR 0.12, 95% CI 0.02‐0.88) less likely to acquire droplet‐transmitted viral infections.

When only the continuous N95 arm was compared against control, the risk of laboratory‐confirmed influenza was significantly lower in continuous N95 arm (RR 0.23 and 95% CI 0.06‐0.93, or 77% efficacy). In the similar analysis, the risk of influenza was also lower in medical mask arm compared to control; however, the difference was not statistically significant (RR 0.81 and 95% CI 0.25‐2.68) arm. Table 5 compares the results of this analysis with the individual studies.

**Table 5:** Results of individual clinical trials and pooled analysis

	Arms	RCT 1 (OR/ RR)	RCT 2 (HR/ RR)	Pooled analysis
CRI	Continuous N95	0.46 (0.19‐1.11)	**0.39 (0.21‐0.71)**	
	Targeted N95	‐	0.70 (0.39‐1.24)	
	Medical masks	0.74 (0.29‐1.88)	Ref	
	Control	Ref	‐	
Influenza like illness	Continuous N95	0.26 (0.06‐1.11)	‐	
	Targeted N95	‐	‐	
	Medical masks	0.49 (0.12‐2.07)	‐	
	Control	Ref	‐	
Laboratory‐confirmed viruses	Continuous N95	**0.43 (0.20‐0.91)**	‐	**0.46 (0.23‐0.91)**
	Targeted N95	‐	‐	0.70 (0.30‐1.67)
	Medical masks	0.84 (0.38‐1.85)	‐	0.78 (0.39‐1.56)
	Control	Ref	‐	Ref
Laboratory‐confirmed influenza	Continuous N95	0.25 (0.06‐1.00)	‐	0.34 (0.10‐1.11)
	Targeted N95	‐	‐	0.46 (0.06‐3.40)
	Medical masks	0.81 (0.25‐2.68)	‐	0.55 (0.16‐1.91)
	Control	Ref	‐	Ref
Laboratory‐confirmed bacterial colonisation	Continuous N95	**0.34 (0.21‐0.56)**	**0.40 (0.21‐0.73)**	**0.33 (0.21‐0.51)**
	Targeted N95	‐	0.70 (0.40‐1.24)	**0.54 (0.33‐0.87)**
	Medical masks	0.67 (0.38‐1.18)	Ref	0.74 (0.48‐1.13)
	Control	Ref		Ref
Droplet‐transmitted infections	Continuous N95	‐	‐	**0.26 (0.16‐0.42)**
	Targeted N95	‐	‐	**0.43 (0.25‐0.72)**
	Medical masks	‐	‐	0.65 (0.41‐1.04)
	Control	‐	‐	Ref

Bold value indicates statistically significant results.

## 4 DISCUSSION

We demonstrated superior clinical efficacy of continuous use of N95 respirator (also known as “airborne precautions”) against infections presumed to be spread by the droplet mode, including influenza. This suggests that transmission is more complex than assumed by traditional classifications, and supports the fact that both large and small droplets are present close to the patient, and that aerosol transmission may occur for presumed “droplet” infections. Respirators are designed to provide respiratory protection through filtration and fit, and properly fitted respirators provide better protection compared to medical masks.^3^, ^4^ We could not demonstrate efficacy of medical masks against any outcome, but the non‐significant trend appeared to be towards protection. Medical masks may well have efficacy,^5^ but if so, the degree of efficacy was too small to detect in this study, and larger studies are needed, given the widespread use of these devices in health care.

The practical implication of this research is illustrated with influenza as a case in point. Droplet and contact are thought to be primary modes of transmission for seasonal influenza; therefore, the World Health Organisation (WHO) and the Centers for Disease Control and Prevention (CDC) guidelines recommend medical masks during routine patient care, while N95 respirators are recommended during procedures in which aerosols may be generated and during other high‐risk situations.^14^, ^15^ However, there is increasing evidence of aerosol transmission of influenza during routine care as well (in the absence of aerosol generating procedures), which may warrant superior respiratory protection.^16^, ^17^ Influenza research is challenging because there is high seasonal variation in activity, and the level of circulating influenza in any given year cannot be predicted when planning RCTs. In addition, a diagnosis of influenza requires the detection of virus from respiratory specimens, or a fourfold rise in serological titres, both of which are highly resource‐intensive and depend on daily subject follow‐up and on optimal timing of specimen collection. For all these reasons, the published studies to date have been unable to determine whether there is a difference in efficacy against influenza infection between medical masks and N95 respirators. This study can therefore usefully inform policies for prevention of influenza.

In the first RCT, compared to medical masks, N95 respirators were found to be protective against CRI, but not against ILI or laboratory‐confirmed influenza.^3^ When compared with the control arm, rates of laboratory‐confirmed virus and bacterial colonisation were significantly lower in N95 arm (Table 5). In the second RCT, continuous use of N95 respirators was associated with lower rates of CRI and laboratory‐confirmed bacterial colonisation compared to the medical mask use.^4^ Pooled analysis of these studies improved the power to analyse other infectious outcomes by intervention and to allow analysis by mode of transmission.

An important finding of this analysis was the efficacy of N95 respirators against droplet‐transmitted infections. Generally, medical masks are considered sufficient for droplet‐transmitted infections such as influenza.^18^ However, this study has demonstrated a clear benefit of using N95 respirators (both continuous and targeted) to protect HCWs against droplet infections and does not show significant protection of medical masks. In the light of these findings, it may be prudent to use respirators when the transmission mode of a disease is unknown or when HCWs exposed to droplet‐transmitted infections with a high‐case fatality rate.^6^ Middle East respiratory syndrome coronavirus (MERS‐CoV) and Ebola virus disease (EVD) are not airborne infections, yet the CDC recommendation of using respirators to protect HCWs recognises the uncertainty around transmission.^19^, ^20^ The CDC initially recommended medical masks for Ebola, but changed their guidelines when US HCWs became infected, amidst unrest and challenges to the prior guidelines.^6^, ^21^ In contrast, the WHO recommends medical masks for MERS‐CoV and Ebola 22, 23 despite having older guidelines for filoviruses which recommended respirators.^24^ There is a need for a more evidence‐based approach to updating guidelines and ensuring consistency between different guidelines.^25^

Our study also demonstrated that, over and above the benefit of continuous use, targeted use of N95 is associated with reduced risk of infection. Many guidelines recommend targeted use,^14^, ^15^, ^26^ and our study supports this practice. However, better protection is achieved through continuous use of respirators. This may be because HCWs cannot always identify situations in which they are at risk, especially in busy clinical settings with a high level of movement of patients and staff in and out of wards.

This study has some limitations. Firstly, the reporting of the results included in Figure 1 is different from the IPD meta‐analysis results. This is due to the uneven distribution of randomisation arms and differing seasonal attack rates between the trials. In Figure 1, these between‐trial differences were not taken into account. The IPD meta‐analysis takes into account of these and gives an unbiased association. Secondly, the control arm in trial 1 was not randomised; however, the risk of bias is less due to similar study setting, outcome measures and participant characteristics. Moreover, whether infection was acquired in the community or the hospital cannot be determined, but the RCT design should result in community exposure being distributed equally across all arms. Finally, we categorised pathogens according to various transmission modes, while certain viruses are transmitted via multiple routes. The pooled data were suggestive of an effect of respirators against influenza, but probably did not have enough statistical power for this outcome. The major strength of this study is the use of the same endpoints, measurements and methods in the two trials, which allowed valid pooling of the data.

## 5 CONCLUSION

It is a long‐held belief in hospital infection control that a mask is adequate for droplet‐transmitted infections. We showed that the use of respirators provides better protection against respiratory infections, even those presumed to be spread predominantly by the droplet mode. The targeted use of a respirator was also effective, whereas no efficacy was demonstrated for medical masks alone. However, the trends suggest some degree of protection from medical masks, and larger studies are required to measure the efficacy of these devices. The superiority of respirators should be reflected in infection control guidelines to ensure the occupational health and safety of HCWs. A growing body of clinical efficacy evidence, including this study, challenges long‐held paradigms about the transmission of infection.

## 6 SUMMARY OF KEY POINTS

1. The data collected during two similar clinical trials conducted in Beijing, China, which examined the same infection outcomes, were pooled
2. We showed that respirators provide superior protection against droplet‐transmitted infections, for which most guidelines recommend masks. These findings challenge the paradigm of infection transmission being simplified to droplet, airborne or contact.
3. For many infections, more than one mode of transmission is possible, and our data suggest that transmission of infections is more complex than suggested by these paradigms.
4. Clinical efficacy data are a higher level of evidence than theoretical paradigms of transmission, and show better protection afforded by respirators.

## COMPETING INTERESTS

All authors have completed the Unified Competing Interest form (available on request from the corresponding author) and declare that; (i) Professor C. Raina MacIntyre: Raina MacIntyre has held an Australian Research Council Linkage Grant with 3M as the industry partner, for investigator‐driven research. 3M have also contributed supplies of masks and respirators for investigator‐driven clinical trials. She has received research grants and laboratory testing as in‐kind support from Pfizer, GSK and Bio‐CSL for investigator‐driven research; (ii) Dr Holly Seale had a NHMRC Australian based Public Health Training Fellowship at the time of the study (1012631). She has also received funding from vaccine manufacturers GSK, bio‐CSL and Saniofi Pasteur for investigator‐driven research and presentations, and (iii) Dr. Abrar Chughtai had testing of filtration of masks by 3M for PhD. The remaining authors declare that they have no competing interests and have no non‐financial interests that may be relevant to the submitted work.

## AUTHORS’ CONTRIBUTIONS

CRM performed the lead investigator, conception and design of the study, analysing the data and manuscript writing; AAC involved in the statistical analysis and drafting of manuscript; BR performed the contribution to the statistical analysis and revision of manuscript; YP, YZ, HS and XW performed the data management and revision of manuscript; QW performed the contribution to design, analysis and revision of study. All authors have read and approved the final version of the manuscript and ensure that this is the case.

## TRIAL REGISTRATION

This is pooled analysis of two clinical trials.

1. First clinical trial—Clinical trial registered with Australian New Zealand Clinical Trials Registry (ANZCTR) (http://www.anzctr.org.au), registration number ACTRN 12609000257268, registered on 13/05/2009.
2. Second clinical trial—Clinical trial registered with Australian New Zealand Clinical Trials Registry (ANZCTR) (http://www.anzctr.org.au), registration number ACTRN 12609000778280, registered on 8/09/2009.
