# Supplementary material for: The efficacy of medical masks and respirators against respiratory infection in healthcare workers
Source: Influenza Other Respir Viruses. 2017 Aug 30;11(6):511–7. doi: 10.1111/irv.12474 (PMC5705692; doi:10.1111/irv.12474)
Supplement: Supplementary file 1 [file IRV-11-511-s001.docx]

**SUPPLEMENT A:**

**TITLE: The efficacy of medical masks and respirators against respiratory infection in health workers**

**Table s1: Categorization of bacteria/virus according to the mode of transmission**

|  | **Primary mode** | **other main modes** |
| --- | --- | --- |
| **Virus** |  |  |
| Adenovirus [1] | Contact | Fecal-oral, droplet |
| Coronavirus [2] | Droplet | Contact |
| Parainfluenza virus [3] | Contact | Droplet |
| Influenza A virus [4] | Droplet | Contact, airborne |
| Influenza B virus [5] | Droplet | Contact |
| Respiratory syncytial virus [6] | Contact | Droplet |
| Rhinovirus A/B [7] | Airborne | Droplet |
| **Bacteria** |  |  |
| Streptococcus pneumoniae [8] | Droplet | contact |
| Haemophilus influenzae [9] | Droplet | contact |
| Bordetella pertussis [10] | Droplet | airborne |
| Chlamydophila pneumoniae [11] | Droplet | contact |
| Mycoplasma pneumoniae [12] | Droplet | contact |

**Reference**

1. Public Health Agency of Canada. Adenovirus [cited 2014 4 November]. Available from: <http://www.phac-aspc.gc.ca/lab-bio/res/psds-ftss/adenovirus-eng.php>.

2. Public Health Agency of Canada. Human coronavirus [cited 2014 4 November]. Available from: <http://www.phac-aspc.gc.ca/lab-bio/res/psds-ftss/coronavirus-eng.php>.

3. Public Health Agency of Canada. Human parainfluenza virus [cited 2014 4 November]. Available from: <http://www.phac-aspc.gc.ca/lab-bio/res/psds-ftss/parainfluenza-eng.php>.

4. Public Health Agency of Canada. Influenza virus type A [cited 2014 4 November]. Available from: <http://www.phac-aspc.gc.ca/lab-bio/res/psds-ftss/influenza-a-eng.php>.

5. Public Health Agency of Canada. Influenza virus type B [cited 2014 4 November]. Available from: <http://www.phac-aspc.gc.ca/lab-bio/res/psds-ftss/influenza-grippe-b-c-eng.php>.

6. Public Health Agency of Canada. Respiratory syncytial virus [cited 2014 4 November]. Available from: <http://www.phac-aspc.gc.ca/lab-bio/res/psds-ftss/pneumovirus-eng.php>.

7. Public Health Agency of Canada. Rhinovirus [cited 2014 4 November]. Available from: <http://www.phac-aspc.gc.ca/lab-bio/res/psds-ftss/rhinovirus-eng.php>.

8. Public Health Agency of Canada. Streptococcus pneumoniae [cited 2014 4 November]. Available from: <http://www.phac-aspc.gc.ca/lab-bio/res/psds-ftss/streptococcus-pneumoniae-eng.php>.

9. Public Health Agency of Canada. Haemophilus influenzae [cited 2014 4 November]. Available from: <http://www.phac-aspc.gc.ca/lab-bio/res/psds-ftss/haemophilus-influenzae-eng.php>.

10. Public Health Agency of Canada. Bordetella pertussis [cited 2014 4 November]. Available from: <http://www.phac-aspc.gc.ca/lab-bio/res/psds-ftss/bordetella-pertussis-eng.php>.

11. Center for Disease Control and Prevention (CDC). Chlamydophila pneumoniae Infection [cited 2014 4 November]. Available from: <http://www.cdc.gov/pneumonia/atypical/chlamydophila.html>.

12. Public Health Agency of Canada. Mycoplasma pneumoniae [cited 2014 4 November]. Available from: <http://www.phac-aspc.gc.ca/lab-bio/res/psds-ftss/myco-pneu-eng.php>.
